# Novel Seleno-Aspirinyl Compound AS-10 Induces Apoptosis, G1 Arrest of Pancreatic Ductal Adenocarcinoma Cells, Inhibits Their NF-κB Signaling, and Synergizes with Gemcitabine Cytotoxicity

**DOI:** 10.3390/ijms22094966

**Published:** 2021-05-07

**Authors:** Deepkamal N. Karelia, Sangyub Kim, Manoj K. Pandey, Daniel Plano, Shantu Amin, Junxuan Lu, Arun K. Sharma

**Affiliations:** 1 Department of Pharmacology, Penn State College of Medicine, 500 University Drive, Hershey, PA 17033, USA; dkarelia@pennstatehealth.psu.edu (D.N.K.); kimx2920@umn.edu (S.K.); pandey@rowan.edu (M.K.P.); dplano@unav.es (D.P.); sga3@psu.edu (S.A.); 2 Penn State Cancer Institute, 500 University Drive, Hershey, PA 17033, USA

**Keywords:** pancreatic cancer, apoptosis, selenium, aspirin, RNA-Seq

## Abstract

Current available therapies for pancreatic ductal adenocarcinoma (PDAC) provide minimal overall survival benefits and cause severe adverse effects. We have identified a novel molecule AS-10, a selenazolidine-bis-aspirinyl derivative, that was two to three orders of magnitude more potent than aspirin and at least one to two orders of magnitude more potent than gemcitabine in inhibiting PDAC cancer cell growth/viability against three PDAC cell lines while sparing mouse embryonic fibroblasts in the same exposure range. In Panc-1 cells, AS-10 induced apoptosis without necrosis, principally through caspase-3/7 cascade and reactive oxygen species, in addition to an induction of G_1_ cell cycle block. Transcriptomic profiling with RNA-seq indicated the top responses to AS-10 exposure as *CDKN1A* (P21Cip1), *CCND1*, and nuclear transcription factor-kappa B (NF-κB) complex and the top functions as cell cycle, cell death, and survival without inducing the DNA damage gene signature. AS-10 pretreatment (6 h) decreased cytokine tumor necrosis factor-alpha (TNF-α)-stimulated NF-κB nuclear translocation, DNA binding activity, and degradation of cytosolic inhibitor of κB (IκB) protein. As NF-κB activation in PDAC cells confers resistance to gemcitabine, the AS-10 combination with gemcitabine increased the in vitro cytotoxicity more than the additivity of both compounds. Overall, our results suggest AS-10 may be a promising drug lead for PDAC, both as a single agent and in combination therapy.

## 1. Introduction

Pancreatic cancer presents mostly as pancreatic ductal adenocarcinoma (PDAC). It is usually detected at an advanced stage due to the lack of specific screening biomarkers and complications from other health conditions that confound an accurate diagnosis. Not only highly loco-regional invasive and metastatic, PDAC possesses a very heavily fibrotic stroma that hinders delivery of systemic chemotherapeutic drugs and is hard to treat, making it the fourth ranked deadliest cancer in the US [1,2]. Gemcitabine (2′-deoxy-2′,2′-difluorocytidine), as a single agent, was for a long time the first-line standard of care chemotherapy drug, providing limited efficacy, with an objective response rate of 8-11% and median survival benefit of 4–7 months [3]. Its combination with several targeted drugs, including those against Ras (farnesyl transferase inhibitors) [4], receptor tyrosine-protein kinase erbB-2 (HER2) [5], matrix metalloproteinases (MMPs) [6], vascular endothelial growth factor (VEGF) [7], and epidermal growth factor receptor (EGFR) [8], was unsuccessful or at best, provided only marginal improvement. For example, gemcitabine + erlotinib (an EGFR inhibitor drug) improved survival by 2 weeks compared to gemcitabine alone (6.2 months (combination) vs. 5.9 months (gemcitabine alone) but was not statistically significant [9]. More recently, a combination of nanoparticle albumen-bound (nab)-paclitaxel + gemcitabine in metastatic PDAC patients significantly improved overall survival, progression-free survival, and response rate but led to increased peripheral neuropathy and myelosuppression rates [10]. The new first-line standard of care therapy for metastatic pancreatic cancer patients, FOLFIRINOX, is a combination regimen of 5-fluorouracil, leucovorin, irinotecan, and oxaliplatin that increases survival superior to gemcitabine, but few patients can tolerate the greater toxicity [11]. Maintenance therapies [12], such as capecitabine (a 5-flurouracil prodrug), have been tested post first-line therapy to cope with such toxicities but with limited success. Due to the complexity of the PDAC tumor microenvironment, so far, all clinical trials with immune-checkpoint inhibitors as monotherapy have yielded poor outcomes and early termination of the studies [13]. Currently, immunotherapy + chemotherapy combination trials are ongoing. Therefore, there exists an urgent unmet clinical need for alternative therapeutics and/or drug combinations to improve overall survival and reduce toxicities.

The NF-κB pathway has been found to be upregulated in many malignancies, including PDAC, and is induced by chemotherapeutic drugs in PDAC [14], thus making it a plausible target for combinational treatments [15]. NF-κB is a complex of two proteins: RelA (also known as P65) and P50. Canonical activation of the NF-κB pathway is stimulated by the binding of a proinflammatory cytokine, such as tumor necrosis factor-alpha (TNF-α), to its receptor, activating the receptor tyrosine kinase cascade to activate the IκB kinase complex (IKK) [16]. The active IKK phosphorylates the inhibitor of κB protein-α (IκBα), promoting its polyubiquitination and proteosomal degradation, and releasing the RelA and P50 complex to translocate to the nucleus to activate target genes involved in cell survival (Bcl-2, Bcl-xL, Mcl-1, etc.), proliferation (cyclin D), and angiogenesis and invasion (VEGF, MMPS, etc.). NF-κB also regulates the expression of a number of growth factors and proinflammatory cytokines [16].

**Figure 1:**
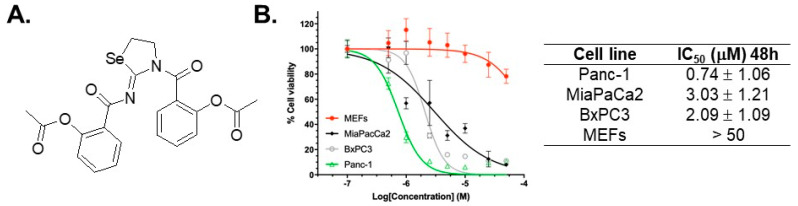
AS-10 selectively inhibited PDAC cell growth. (**A**) Structure of AS-10. (**B**) Cell number/viability MTT assay and IC_50_ chart. Panc-1, MiaPaCa2, BxPC3, and MEF cells were treated with AS-10 (concentration range 0–50 µM) for 48 h. Curves by non-linear regression using variable slope equation in GraphPad prism software show the cell number relative to the respective DMSO control. Data represented as mean ± SD. IC_50_ data represented as mean ± SD of three independent experiments.

The non-steroidal anti-inflammatory drug (NSAID) acetylsalicylic acid (ASA, commonly known as Aspirin) has received much attention for its chemo-preventive ability in different cancers, including colorectal cancer (CRC) and PDAC [17,18]. A literature review has associated high doses of ASA use with decreased risk for PDAC [19] but significant gastrointestinal (GI) bleeding toxicity [20]. Efforts have been made to optimize ASA for better efficacy and less bleeding toxicity [21,22,23]. In cell culture and animal models, organoselenium compounds possess promising anticancer properties, stimulating interest in designing selenium (Se) containing small molecules [22,24,25,26]. The most commonly described mechanisms by which Se compounds exert their anticancer ability are induction of ROS or quenching of ROS; inhibition of different pro-survival proteins (like Bcl-xL and Survivin); induction of apoptosis; inhibition of angiogenesis; and modulation of AKT, COX, P38, and NF-κB-signaling pathways [27]. Furthermore, Se compounds have been shown to protect against gastric toxicity induced by the COX inhibitor drug indomethacin [28], supporting combination of NSAIDs and Se to design novel Se-NSAID hybrid compounds [22]. In continuation of our pursuits to develop Se-NSAID compounds, we have now identified a novel compound, AS-10, having a cyclic selenazolidine ring substituted by two aspirinyl moieties (Figure 1A). Herein, we characterized the cell cycle and cell death responses of PDAC cells to AS-10 and assessed any involvement of ROS. We employed transcriptomic profiling of the global gene expression changes induced by AS-10, highlighting the NF-κB pathway as well as transcriptional-level controls of cell cycle arrest and apoptosis. We investigated the specific impact of AS-10 on NF-κB signaling and combination with gemcitabine in vitro to synergize cytotoxicity.

## 2. Results

### 2.1. Selectivity of AS-10 to Inhibit PDAC Cell Growth Over Non-Malignant Cells

**Figure A1:**
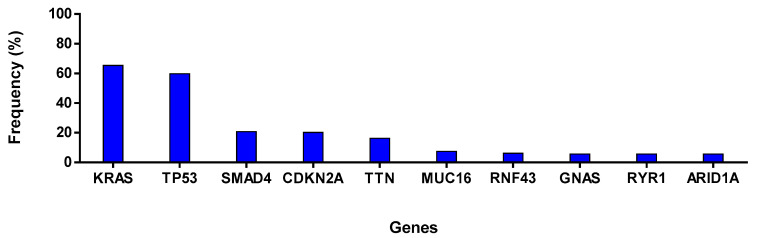
The top most frequently mutated genes in PDAC patients [TCGA, Pancreatic Atlas; *n* = 175] dataset.

The MTT cell viability (number) assay was used to assess the growth inhibitory and cytotoxic impacts of AS-10 on PDAC cell lines. Because *KRAS* and *TP53* are the top mutated genes in the clinical PDAC specimens (Figure A1), we chose two cell lines with mutant *KRAS* (Panc-1, MiaPaCa2) and one with wild-type (WT) *KRAS* (BxPC3), all possessing mutant *TP53* (Table S1). Our data showed that AS-10 inhibited cell viability of PDAC cells compared to the DMSO vehicle control, with an IC_50_ in the sub-micromolar to low µM range after 48 h of exposure (Figure 1B). Their *KRAS* mutational status did not correlate with the growth inhibitory response as Panc-1 cells manifested as the most responsive and MiaPaCa2 the least among the three cell lines. In contrast to the three PDAC cell lines, AS-10 treatment of non-cancerous mouse embryonic fibroblast cells (MEFs) (Figure 1B) did not affect the MTT readout significantly in the same exposure range and duration. Taken together, AS-10 exerted a reasonable selectivity to inhibit growth and/or kill PDAC cells. Because ASA required milli-molar concentration exposure to affect the proliferation and survival of PDAC cells [29], the novel compound was two to three orders of magnitude more potent than ASA.

### 2.2. AS-10 Induced Apoptotic Cell Death in Panc-1 Cells

**Figure 2:**
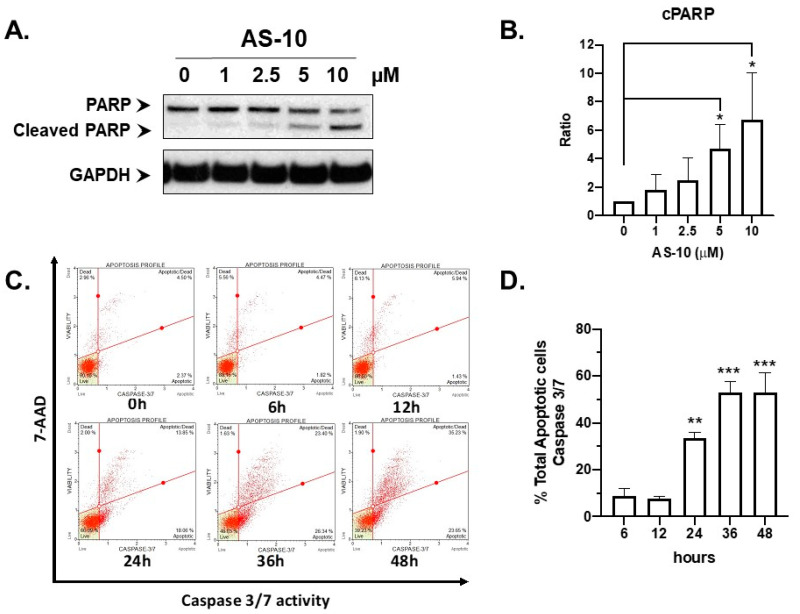
AS-10 induced caspase activation and apoptosis in Panc-1 cells. (**A**) Western blot analysis of PARP cleavage activation. Panc-1 cells were treated with different concentrations of AS-10 for 24 h before whole-cell lysates were prepared for blotting with PARP antibody. GAPDH protein was probed as the loading control. (**B**) Densitometry analysis of cleaved PARP (cPARP) normalized to the house keeping protein evaluated by Image J. Data represented as the means ± SD of three independent experiments, * (*p* < 0.02). (**C**) Illustrative examples of Muse caspase 3/7 activity time course outcomes. Panc-1 cells were treated with 5 µM AS-10 for the indicated duration. Bottom-left quadrant represents healthy cells; bottom-right quadrant represents early apoptotic cells (Caspase 3/7 (+) and 7-ADD (-)); top-right quadrant represents late apoptotic/dead cells (Caspase 3/7 (+) and 7-ADD (+); Top-left quadrant represents cells that died of necrosis (Caspase 3/7 (-) and 7-ADD (+). (**D**) Total apoptotic cells (early + late apoptotic cells) measured by Muse caspase 3/7 activity assay as a function of exposure duration to 5 µM AS-10. Data represented as the means ± SD of three independent experiments, ** (*p* < 0.01), *** (*p* < 0.001).

Panc-1 cells have the most clinically relevant mutations among the three lines examined (mutant *KRAS* and mutant *TP53* and homozygous deletion of *CDKN2A* gene coding for P16Ink4a involved in cell cycle control) [30]. Therefore, we chose Panc-1 cells for subsequent work to characterize the cell death and growth responses and explore molecular signaling mechanisms. To assess cell death, we exposed Panc-1 cells to increasing concentrations of AS-10 for 24 h and used Western blot to detect the cleavage of poly ADP ribose polymerase (PARP), the best-known target of the activated death executioner caspase-3/7 in cells undergoing apoptosis [31]. Figure 2A,B showed a clear concentration-dependent increase of PARP cleavage.

By the Muse caspase 3/7 activity/7-ADD cell membrane integrity assay (Figure 2C,D), AS-10-exposed Panc-1 cells showed significantly increased caspase3/7-positive early (Figure 2C, lower right sector) and late-stage (Figure 2C, upper right sector) apoptotic cells at 24 h and plateaued between 36 and 48 h (Figure 2D). AS-10 did not induce appreciable necrotic cells that were permeable to 7-ADD detectable in the upper-left sector (Figure 2C). Therefore, AS-10 likely induced apoptosis via caspase 3/7 executioners to cleave their death target proteins, without necrosis.

### 2.3. N-Acetyl Cysteine (NAC) Attenuated AS-10-Induced Apoptosis of Panc-1 Cells

**Figure 3:**
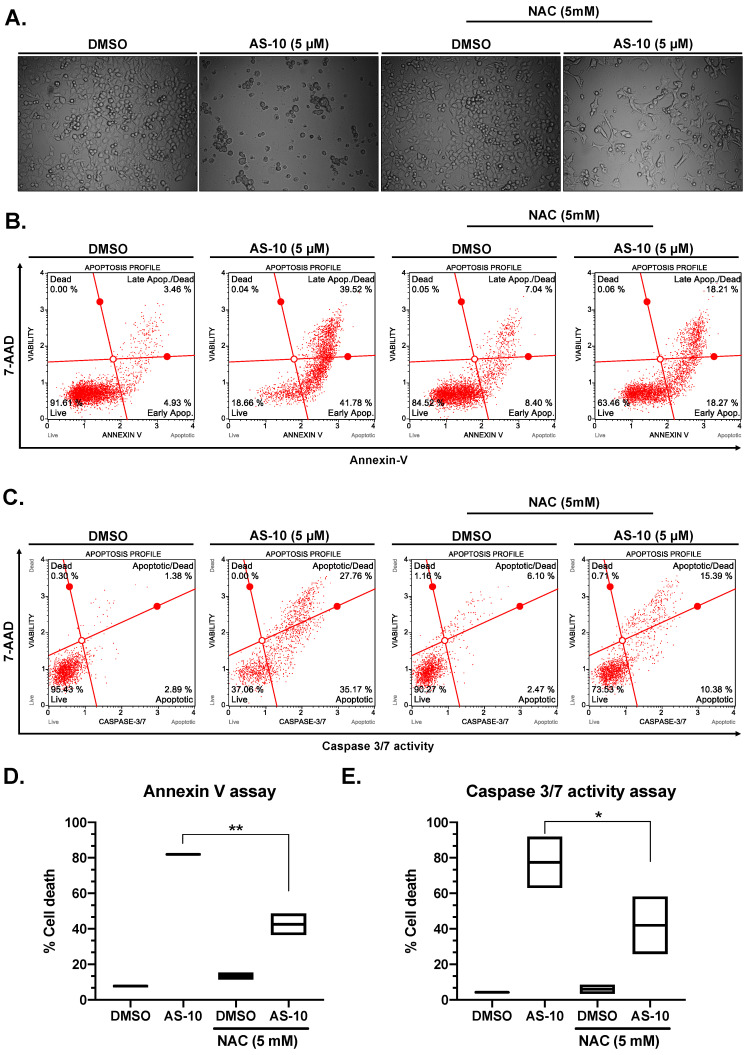
N-acetyl cysteine (NAC) attenuated AS-10-induced apoptosis of Panc-1 cells. Panc-1 cells were pre-treated with NAC for 2 h (in the NAC group) and then co-treated with DMSO or AS-10, for 48 h. After 48 h, cells were subjected to (**A**) light microscopy; (**B**) Muse Live/dead Annexin V assay; and (**C**) Muse Caspase 3/7 activity assay. (**B**,**C**) Representative histograms show four quadrants for estimating live, necrotic, and apoptotic death fractions: bottom-left quadrant (live cells (7-ADD (-), Annexin V (-) or Caspase 3/7 (-))); bottom-right quadrant (Early apoptotic (7-ADD (-), Annexin V (+) or Caspase 3/7 (+))); top-right quadrant (Late apoptotic/dead cells (7-ADD (+), Annexin V (+), Caspase 3/7 (+))); top-left quadrant (necrotic (7-ADD (+), Annexin V (-), Caspase 3/7 (-))).(**D**,**E**) Total apoptotic cells (early + late apoptotic cells) measured by Muse Annexin V assay (**D**) and Muse caspase 3/7 activity assay (**E**). Data represents mean ± SD * (*p* < 0.05); ** (*p* < 0.02).

Cancer cells have higher energy requirements and often generate higher levels of ROS compared to normal cells, making them more susceptible to ROS-mediated cell death [32]. To determine if ROS played a role in AS-10-mediated death of Panc-1 cells, we pre-treated them with/out NAC for 2 h, followed by co-treatment with AS-10 and measured the AS-10-induced cell death at 48 h of exposure with a number of assays. Morphologically, under a phase contrast microscope, the number of cells showing apoptotic features (single floaters) was fewer in wells treated with the combination of NAC with AS-10 than in those with AS-10 treatment alone and increased the proportion of adherent cells (Figure 3A). Pre-treatment with NAC, followed by co-treatment with AS-10 decreased the total apoptotic cells compared to the vehicle with AS-10, detected by the Muse Annexin-V/7-ADD assay (Figure 3B,D, from 82.7% to 48.7%) and Muse Caspase 3/7/7-ADD activity assay (Figure 3C,E, 92% to 58.2%). However, NAC did not restore the growth of AS-10-exposed cells (Figure 3A).

### 2.4. AS-10 Induced G_1_/G_0_ Cell Cycle Arrest in Panc-1 Cells

**Figure 4:**
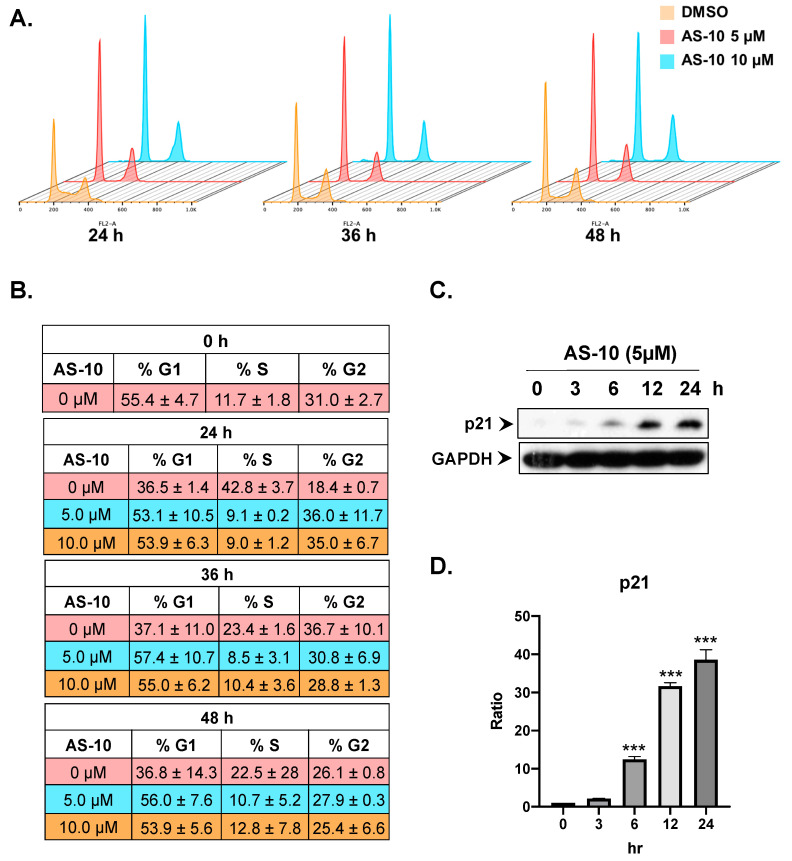
AS-10 induced G1 cell cycle arrest of Panc-1 cells. (**A**,**C**) Flow cytometric assessment of the effect of AS-10 on “synchronized” Panc-1 cell cycle progression after release from 72-h serum starvation block. The cells were treated with DMSO or AS-10 at either 5 or 10 µM for the prescribed duration and were harvested, fixed, and stained with PI for flow cytometry for enumerating G1 (first peak), S (Valley between first and second peak), and G2/M (Second peak). (**B**) Quantification of different cell cycle phases at different time points (Mean ± SD). (**C**) Western blot analysis (30 μg of protein was used for each sample) of P21Cip1 cell cycle inhibitory protein of asynchronous Panc-1 cells treated with 5 µM AS-10 for varying durations as indicated. GAPDH was probed as the loading control. (**D**) Densitometry analysis of P21Cip1 normalized to housekeeping protein evaluated by Image J. Data represented as the means ± SD of three independent experiments, *** (*p* < 0.002).

To examine the effects of AS-10 on cell cycle progression, we deprived the Panc-1 cells of fetal bovine serum (FBS) for 72 h in order to enrich/synchronize them to G_1_/G_0_, designated as 0 h, and then stimulated them with FBS to resume cycling in the absence vs. presence of AS-10. The cell cycle distribution was assessed by flow cytometry at different time points. As shown in Figure 4A,B, AS-10-treated cells were blocked in the G_1_/G_0_ phase of the cell cycle as they failed to move into the DNA synthesis phase (S phase) for the duration of the 48 h exposure.

Consistent with G_1_ block, AS-10 exposure of asynchronous Panc-1 cells increased the abundance of CDK inhibitory protein P21Cip1 (Figure 4C,D), in a time-dependent manner, starting as early as 6 h. Subsequent RNA-seq in the next section revealed a substantial difference in the regulation of the key CDKI protein and additional cell cycle regulatory molecules affected by AS-10 exposure.

### 2.5. Transcriptomic Profiling of AS-10 Effects on Panc-1 Cells by RNA-seq

**Figure 5:**
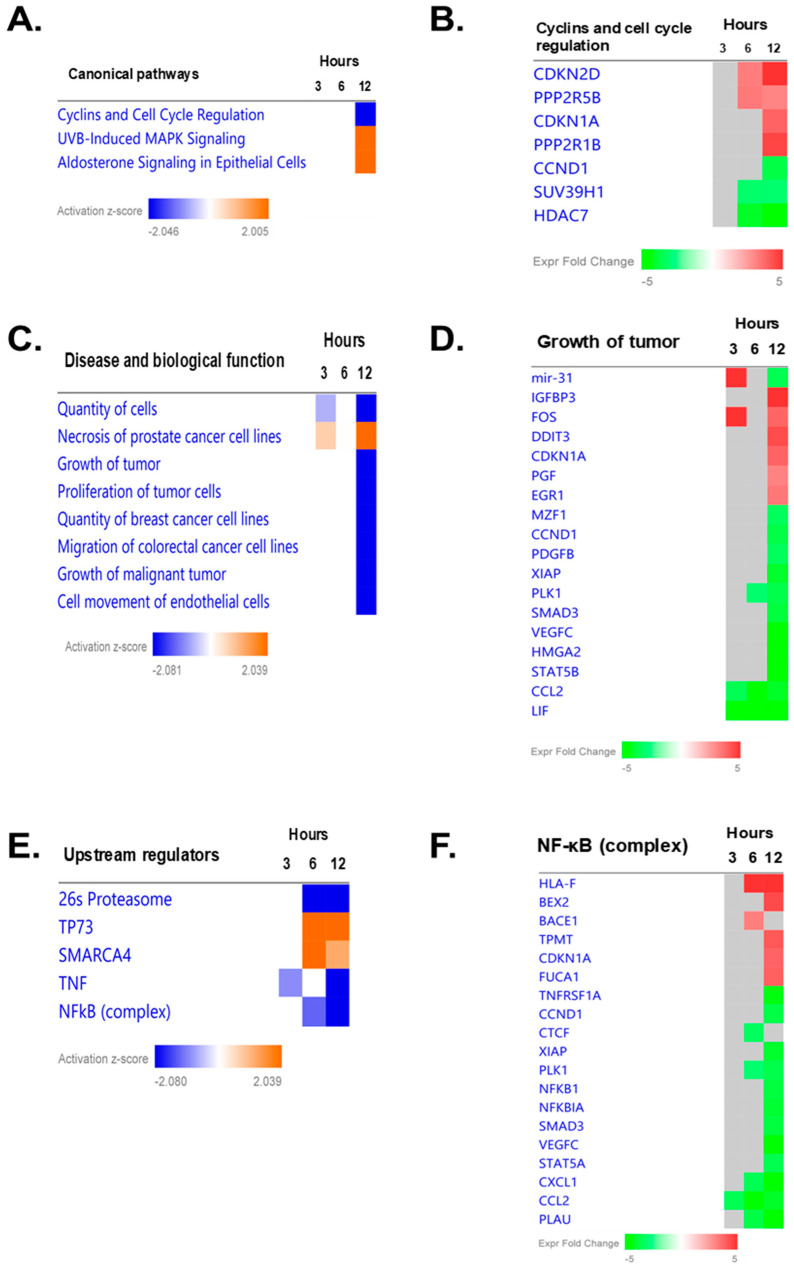
Ingenuity pathway analyses (IPA) of gene expression changes in Panc-1 cells. (**A**) IPA canonical pathways. (**B**) Heatmap of genes in the top canonical pathway (cyclins and cell cycle regulation). (**C**) IPA disease and biological functions. (**D**) Heatmap of genes in the growth of tumor disease and biological function. (**E**) Top 5 IPA upstream regulators. (**F**) Heatmap of genes in NF-κB complex, which displayed a strong time dependence. (**A**,**C**,**E**) The canonical pathways, the disease and biological functions, and the upstream regulators were ranked by Z-scores and the Benjamini–Hochberg (**B**–**F**) *p*-value score of the enrichment score. The color scheme and bar are based on activation Z-scores, with activation in orange, inhibition in blue, and undetermined direction in white. (**B**,**D**,**F**) The heatmaps showing differentially expressed genes at 3, 6, and 12 h, related to the canonical pathways, the disease and biological functions, and the upstream regulators, respectively. Color bar: expression fold change relative to matched DMSO control.

To profile early global gene expression changes induced by AS-10 exposure preceding cell cycle arrest and cell death in Panc-1 cells, we next performed RNA-seq by comparing the paired vehicle (DMSO) and AS-10 (5 µM)-treated Panc-1 cells from 3, 6, and 12 h, i.e., time points before significant apoptosis was detectable. We utilized IPA comparison analysis among 3, 6, and 12 h to identify pathways, functions, and upstream regulators impacted by the gene expression changes at different time points. First, IPA canonical analysis predicted the downregulated *cyclins and cell cycle regulation* at 12 h compared with 3 and 6 h (Figure 5A). RNA-seq data indicated key genes involved in *G*_1_*/S cell cycle checkpoint regulation* from the IPA canonical pathway (G_1_/G_0_ phase currently not available on the IPA canonical pathway) (Figure 5B and Tables S2 and S3). *CDKN1A* (coding P21Cip1) and *CDKN2D* (coding P19Ink4d) were increased and *CCND1* and *CDK6* were decreased in a time-dependent manner (Table S2). Suppression of *FOSL1* and *MYC* was noteworthy for the early response as early as 3 h and for persistence through 12 h. The analysis also predicted additional upregulated signaling changes (Figure 5A). For example, *UVB-induced MAPK signaling* may include stress genes and the *FOS* gene (JNN partner), without increasing DNA damage/P53 marker genes, such as *ATM, ATR, BAX, BID, GADD45A/B, PCNA*, and *TP53* (Table S2). The time-dependent increase of *CAPN5 transcript expression* (Table S2) known to be involved in apoptosis of neuron cells [33] suggested a calcium-driven signaling to caspase-9 apoptosis, likely related to mitochondria permeability transition.

Next, we investigated the biological functions related to the outcomes of the canonical pathways using IPA disease and biological function analysis. This predicted the inhibition of *cancer cell quantity, growth, proliferation, and migration/motility* and activation of *cell death (necrosis)* (Figure 5C). The heatmap of *tumor growth* (overlapping with many genes in the *proliferation of tumor cells*; data not shown) indicated that the genes were associated with inhibition of the cell cycle, proliferation, and angiogenesis; immunoregulatory and inflammatory processes; and the antiapoptotic process (Figure 5D).

We then carried out IPA upstream regulator analysis for those regulating the canonical pathways and the biological functions. The top five predicted upstream regulators were 26s proteasome, TP73 (P53 analog), SMARCA4, TNF (inflammation), and NF-κB complex (Figure 5E). The 26s proteasome is associated with the degradation of abnormal and damaged proteins, cell cycle regulators, and oncogenes and tumor suppressors [34]. TP73 encodes a member of the p53 family of transcription factors involved in cellular responses to stress and development, cell cycle regulation, and apoptosis [35]. This could be particularly relevant in the Panc-1 cells with nonfunctional mutant P53. SMARCA4, a central component of the SWI/SNF chromatin-remodeling complex, functions in the regulation of transcription, cell cycle, and DNA replication [36]. TNF, a critical mediator of immune and inflammatory responses, is involved in the regulation of cell cycle, proliferation, differentiation, and apoptosis [37]. As described in the Introduction, NF-κB complex controls transcription of DNA, cytokine production, and cellular behaviors, including inflammatory responses, cell cycle, growth, survival, and apoptosis [16]. All the upstream regulators are commonly associated with regulation of the cell cycle and proliferation. The gene heatmap of NF-κB signaling involved inhibition of the cell cycle, growth, proliferation, and angiogenesis and immunoregulatory and inflammatory responses (Figure 5F, Table S2). In particular, *NFKBIA* (IκBα) *NFKB1* (P50) and another NF-κB subunit gene *RELB* were decreased by AS-10 in a time-dependent manner (Table S2).

**Figure 6:**
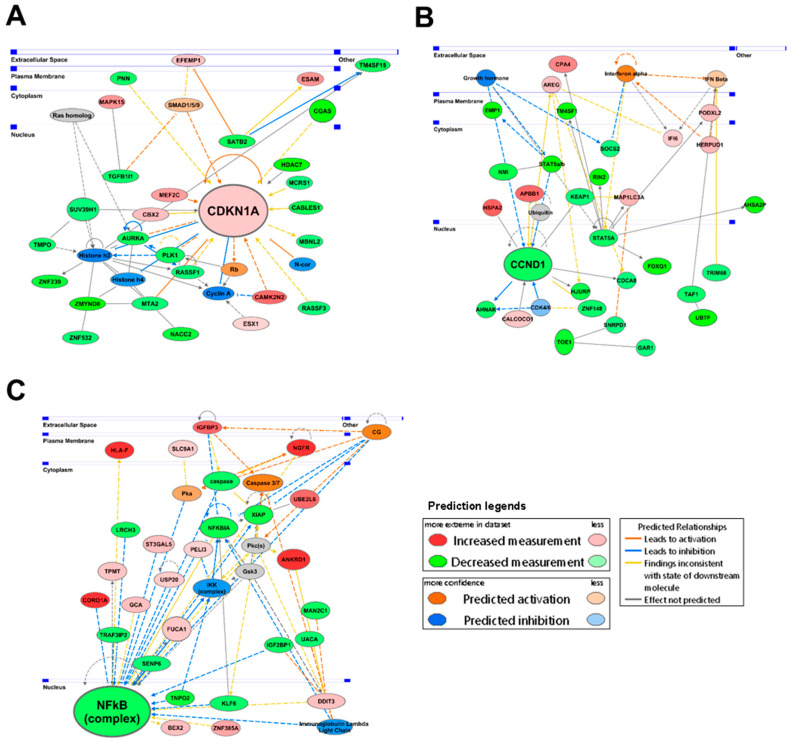
Top 3 IPA-determined networks in Panc-1 cells affected by 5 µM AS-10 at 12 h. (**A**) Network 1 displaying interactions among genes related to cell cycle, growth, and proliferation. (**B**) Network 2 displaying interactions among genes related to embryonic and organ development. (**C**) Network 3 displaying interactions among genes related to cancer, cell death and survival, and organismal injury and abnormalities. Central node molecules (CDKN1A, CCND1, and NF-κB) linking multiple interacting genes were enlarged. The network overlaid with IPA Molecule Activity Predictor displayed the prediction of molecular changes. Red genes were significantly upregulated; green genes were downregulated; grey genes were not significantly changed. The colors of the predicted molecular changes are based on the activation Z-scores, with activation in orange and inhibition in blue.

Using IPA network analysis, we further evaluated interacting molecular networks of the differentially expressed genes following AS-10 treatment at 12 h to identify central molecular targets, functions, and their association with the results of the IPA canonical pathway, disease and biological functions, and upstream regulator analyses. The highest scoring networks affected by AS-10 treatment at 12 h were cell cycle, growth, and proliferation (Figure 6A); embryonic and organ development (Figure 6B); and cancer, cell death and survival, and organismal injury and abnormalities (Figure 6C). CDKN1A, CCND1, and NF-κB were key central nodes in the respective network pathways (Figure 6A–C).

### 2.6. AS-10 Inhibited Activation of NF-ĸB by the Proinflammatory Cytokine TNF-α in Panc-1 Cells

**Figure 7:**
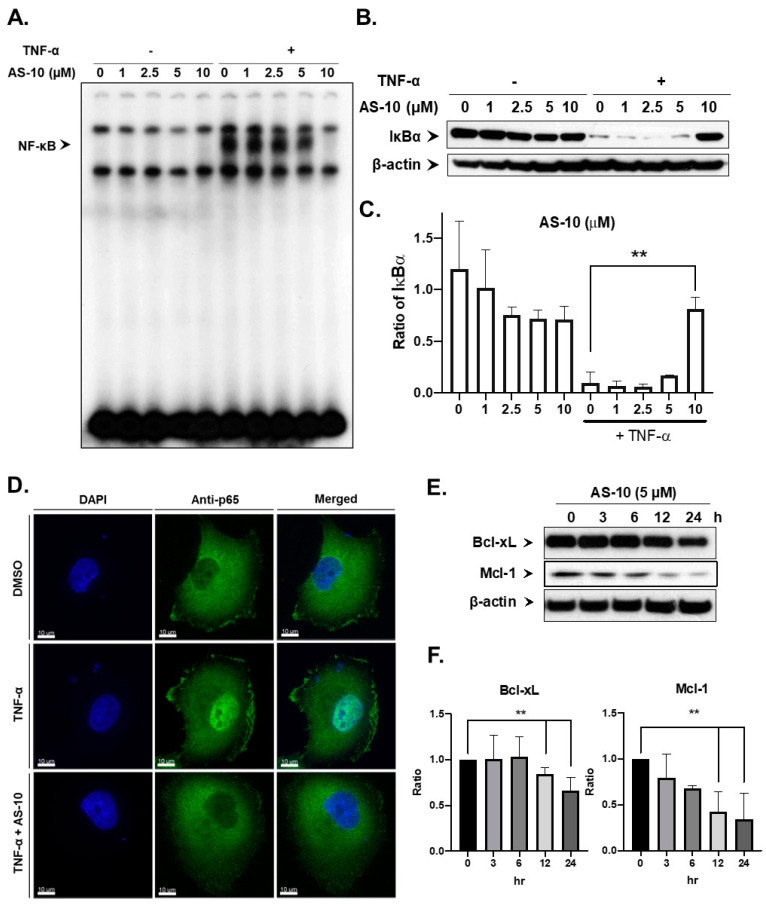
AS-10 inhibited NF-κB signaling in Panc-1 cells. (**A**) NF-κB DNA binding EMSA for nuclear lysates of Panc-1 cells treated with AS-10 at the indicated concentrations for 6 h, and without or with TNF-α stimulation (100 ng/µL, 3.9 nM) for an additional 30 min. (**B**) Western blot (30 μg of protein was used for each sample) of cytosolic fraction of AS-10-treated Panc-1 cells in (**A**) probed with antibody against IκBα. β-Actin was probed as the loading control. (**C**) Densitometry analysis of cleaved IκBα normalized to housekeeping protein evaluated by Image J. Data represented as the means ± SD of three independent experiments, ** (*p* < 0.02) (**D**) Immunocytochemistry fluorescence detection of NF-κB P65 protein in Panc-1 cells seeded on a glass coverslip and treated with 10 µM AS-10 for 6 h and stimulated with TNF-α (100 ng/mL) for an additional 30 min. Resultant cells were fixed and probed with antibody against P65 protein (green). DNA dye DAPI (blue) was used to stain the nucleus. (**E**) Western blot (30 μg of protein was used for each sample) time course analysis of NF-κB downstream targets MCl-1 and Bcl-xL in Panc-1 cells treated with 5 µM AS-10 for the indicated durations (whole cell lysate). β-Actin was probed as the loading control. (**F**) Densitometry analysis of Bcl-xL and Mcl-1 normalized to housekeeping protein evaluated by Image J. Data represented as the means ± SD of three independent experiments, ** (*p* < 0.02).

**Figure A2:**
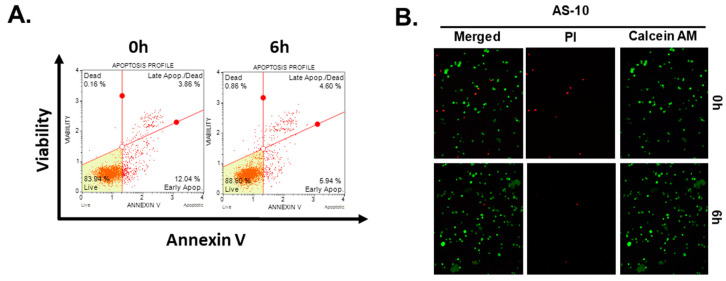
Cell death of Panc-1 cells treated with 10 µM AS-10 required longer than 6 h of exposure. (**A**) Live and dead Annexin V assay. Bottom-left quadrant represents healthy cells; bottom-right quadrant represents early apoptotic cells (Annexin V (+) and 7-ADD (-)); top-right quadrant represents late apoptotic/dead cells (Annexin V (+) and 7-ADD (+); Top-left quadrant represents cells that died of necrosis (Annexin V (-) and 7-ADD (+). (**B**) Fluorescence microscopy Live and Dead analysis using calcein-AM and ethidium bromide. Green indicates live cells and red indicates dead cells.

Given the known importance of NF-κB signaling in PDAC, especially Panc-1 cells, the RNA-seq analysis results prompted us to investigate the ability of AS-10 to inhibit the NF-κB pathway stimulated by the inflammatory cytokine TNF-α. Panc-1 cells were pre-treated with AS-10 for 6 h and then stimulated with/out TNF-α for an additional 30 min for the preparation of nuclear vs. cytosolic fractions. The nuclear lysates were subjected to EMSA using P^32^-labelled canonical NF-κB binding oligonucleotide duplex, while cytosolic lysates were analyzed by Western blot for detection of IκBα degradation. As shown in Figure 7A, treatment of AS-10 decreased NF-κB DNA binding in a concentration-dependent manner. In addition, AS-10 treatment inhibited the degradation of cytosolic IκBα stimulated by TNF-α (Figure 7B,C), in a pattern reciprocal to the DNA binding readout (Figure 7A). The immunocytochemistry assay confirmed that AS-10 pre-treatment prevented the TNF-α-stimulated translocation of P65 protein (subunit of NF-κB) from the cytoplasm to the nucleus (Figure 7D). To confirm that the effects of AS-10 on the NF-κB pathway preceded cell death, we analyzed the AS-10-treated Panc-1 cells for 6 h by not only Annexin V early apoptosis staining/7-ADD assay (Figure A2A) but also a calcein-live and dead (measured by propidium iodide staining) assay (Figure A2B). Neither assay detected an increase in dead cells at this early time point, congruent with the time course of caspase cleavage activation (Figure 2B).

Consistent with inhibition of NF-κB signaling, Western blot detected decreased expression of proteins, such as Bcl-xL and Mcl-1, which are known transcriptional targets of NK-κB, as early as 12 h (Figure 7E,F). RNA-seq also detected decreased Bcl-xL gene (*BCL2L1*), and other BCL-2 gene family genes (*BCL9L, BCL2L2*) while not affecting *MCL1* at the transcript level (Table S2). These proteins regulate mitochondria membrane integrity; therefore, it could suggest the involvement of upstream intrinsic caspase signaling by AS-10.

### 2.7. AS-10 Potentiated the Cytotoxic Effects of Gemcitabine In Vitro

**Figure 8:**
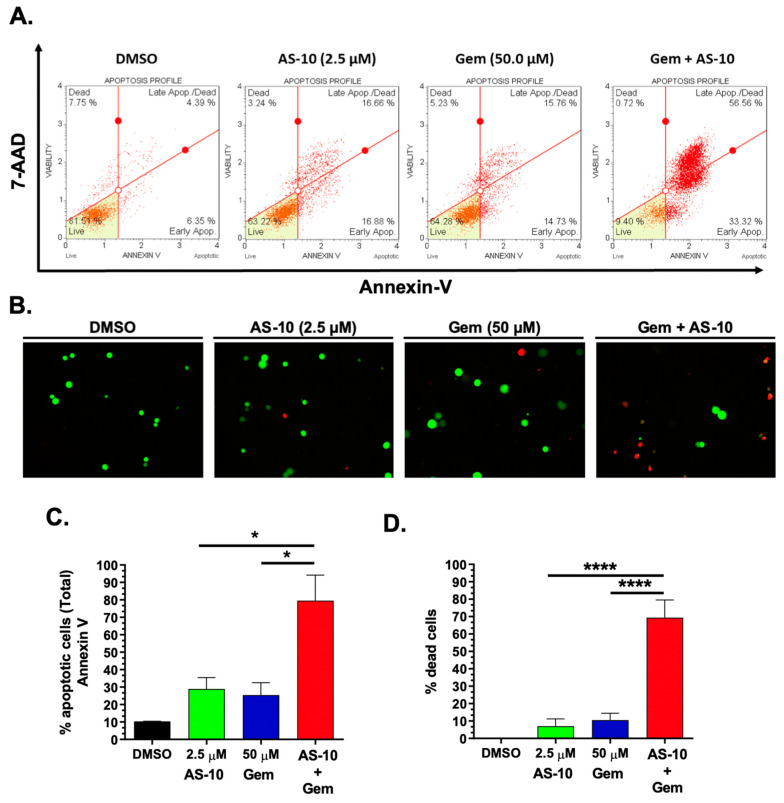
AS-10 potentiated the cytotoxic effects of gemcitabine on Panc-1 cells. Panc-1 cells were treated with AS-10 and/or gemcitabine (Gem) for 48 h and were analyzed by: (**A**,**C**) Muse Live/dead Annexin V assay: Histograms show four quadrants: bottom-left quadrant (Healthy cells (7-ADD (-), Annexin V (-))); bottom-right quadrant (Early apoptotic (7-ADD (-), Annexin V (+))); top-right quadrant (Late apoptotic/dead cells (7-ADD (+), Annexin V (+))); top-left quadrant (necrotic (7-ADD (+), Annexin V (-))); (**B**,**D**) Live/dead calcein-AM and ethidium bromide staining: Microscopic fluorescence images of calcein-AM-stained live cells (green color) and ethidium bromide-stained dead cells (red color); (**C**,**D**) Quantification of total apoptotic cells and total dead cells obtained from (**A**,**B**), respectively. Data represents mean ± SD * (*p* < 0.05); **** (*p* < 0.0001).

**Figure A3:**
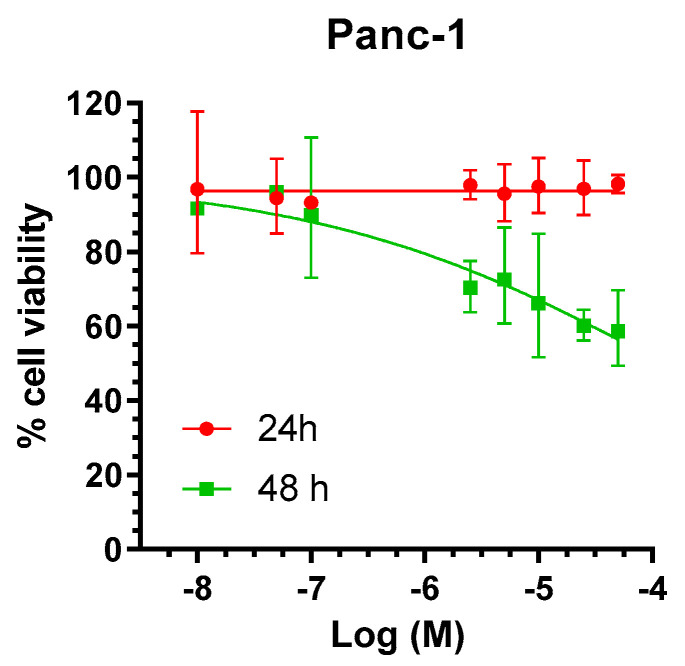
Cell viability of Panc-1 cells in response to increasing Gemzar (gemcitabine) exposure concentrations. Growth curves were plotted by non-linear regression using the variable slope equation in GraphPad prism software. Data represented as mean ± SD.

Activation of the NF-κB pathway is a known mechanism in PDAC cells for resistance towards gemcitabine [38,39,40]. Therefore, we hypothesized that AS-10, by inhibiting the NF-κB pathway and activating intrinsic caspase cascade, would synergize with gemcitabine to kill PDAC cells. In Panc-1 cells, the IC_50_ for gemcitabine was estimated to be higher than 120 µM after 48 h of exposure (Figure A3). Indeed, the combination of the sub-apoptotic concentration of AS-10 (2.5 µM) and gemcitabine (50 µM) resulted in a greater than additive apoptotic response, per independent methods, after 48 h of treatments (Figure 8): Muse Annexin V assay (Figure 8A) and microscopy Live/Dead assay using calcein-AM and ethidium bromide (Figure 8B).

## 3. Discussion

Using clinically relevant PDAC cell lines with respect to the mutational status of oncogene *KRAS*, tumor suppressor *TP53, CDKN2A*, and *SMAD4/DCP4*, our current work established growth suppression and cytotoxicity of AS-10 in cell culture experiments (Figure 1B) that were two to three orders of magnitude more potent than ASA and at least one to two orders of magnitude more potent than gemcitabine (Figure A3). For reference, ASA has been reported to inhibit the growth of PDAC cells at exposure concentrations in the 5–10-mM range [41]. In Panc-1 cells, the IC_50_ for gemcitabine was estimated to be greater than 120 µM after 48 h of exposure (Figure A3). The lack of growth inhibitory and cytocidal activity on normal MEFs by AS-10 (Figure 1B) at exposure concentrations efficacious in the PDAC cell lines supported a reasonable degree of selectivity towards malignant cancer cells. Although based on three PDAC cell lines, all carrying mutant *TP53*, their *KRAS* mutational status did not correlate with the growth inhibitory response to AS-10.

In terms of specific cellular responses in Panc-1 cells, AS-10 induced caspase-mediated apoptosis (Figure 2 and Figure 3) in addition to a persistent G_1_ cell cycle arrest (Figure 4). With respect to apoptotic death signaling, our data suggested that AS-10 treatment led to the activation of caspase-3/7 and cleavage of PARP (Figure 2). Cancer cells show high basal ROS levels because of increased mitochondrial activity as compared to the normal parent cells. Hence, ROS-inducing agents have been proposed to kill cancer cells selectively over normal cells by increasing the amount of ROS enough to tip the balance towards cell death [42]. With NAC co-treatment, the apoptosis execution was attenuated but not blocked (Figure 3), supporting an involvement of ROS in the apoptosis signaling. The activation of the caspase cascade in AS-10-exposed cells suggested the plausibility of mitochondrial transmembrane potential loss and electronic transport chain leakage for the ROS. As gene expression changes did not implicate DNA damage, ROS generation was not likely the primary mediator of AS-10 cellular activities but an associated consequence of apoptosis execution. The type and source of ROS should be further investigated. The time-dependent activation of *CAPN5* (encoding Calpain-5) transcription by AS-10 exposure suggested potential involvement of this calcium-dependent protease to intrinsic caspase signaling, likely related to mitochondria calcium release, and should be further elucidated using siRNA knockdown or CRISPR gene editing technology in the future. Additionally, future investigations using seahorse equipment and flowcytometry techniques (JC-1 assay) could reliably determine the effects of AS-10 on mitochondrial potential and shed insights into the mechanism of action of AS-10. 

For G_1_ cell cycle arrest, we found upregulated CDK inhibitory protein P21Cip1 in a time-dependent manner (Figure 4C,D and Figure 5 and Table S2). Whereas DNA damage is known to activate P21Cip1 through a P53-dependent transcriptional mechanism, this *CDKI* can also be upregulated by P53-independent mechanisms [43]. RNA-seq (Figure 5, Table S2) revealed time-dependent activation of *CDKN1A* gene expression (P21Cip1) by AS-10 exposure without increasing the transcript levels of the mutant *TP53* and its canonical targets *BAX, BID, GADD45A/B*, and *PCNA* or P53 upstream kinases encoded by *ATM* and *ATR,* indicative of DNA damage and repair responses (Table S2), further supporting a P53-independent transcriptional upregulation of *CDKN1A*/P21Cip1. The predicted suppression of 26S proteosome signaling (Figure 5E) could be a reasonable mechanism for a post-translational effect that could also lead to P21Cip1 degradation, which has been suggested as the cause of ASA’s ability to increase P21Cip1 expression [44,45]. ASA has been known to induce expression of cell cycle suppressor proteins [44,45] and to increase histone acetylation [46]. Furthermore, literature reports suggest that histone deacetylase (HDAC) inhibitors, by increasing histone acetylation to increase chromatin accessibility and recruitment of gene expression machinery near the promoter site, can increase P21Cip1 expression in *TP53* mutant cells [47,48]. AS-10 exposure indeed affected a number of HDACs in opposing directions (Table S2), the net balance of which may be involved in regulating *CDKN1A*/P21Cip1 expression by promoting acetylation of histones at the promoter regions. The role of these CDKIs in cell cycle arrest induction by AS-10 should be further elucidated using siRNA knockdown or CRISPR gene editing technology. In addition to the differential CDKI regulations, RNA-seq detected persistent reduction of some CDK and cyclins as well as FOS family members (not Jun family), MYC, and E2F/RB signaling molecules (Tables S2 and S3). Ectopic overexpression approaches for these molecules will facilitate the elucidation of their contributions to the cellular responses observed in the AS-10-exposed cells.

RNA-seq analysis (Figure 5) predicted NF-κB signaling regulation as one of the central nodal activities, aside from inhibition of cell cycle progression and increased cell death. Our follow-up experiments confirmed AS-10 pretreatment blocked inflammatory cytokine TNF-α induction of NF-κB nuclear translocation, decreased NF-κB DNA binding to the canonical target, and inhibited cytosolic IκB degradation (Figure 7). Both ASA- and Se-containing compounds have been documented to inhibit the activity of IKK kinase, by either competing with ATP at the activation site or by covalently binding and inhibiting the activation loop [49]. It has also been reported that acetylation of threonine inside the IKK activation loop can inhibit its activity [50]. Since AS-10 contains a selanazolidine ring flanked by two aspirinyl units, these acetyl groups could acetylate IKK in a similar manner. Additionally, the failure of AS-10-exposed cells to degrade cytosolic IκBα induced by TNF-α suggested that either IKK kinase activity or proteasome activity may have been inhibited by AS-10, as RNA-seq predicted.

The activation of the NF-ĸB pathway is a known mechanism through which PDAC cells develop resistance to gemcitabine [40]. Inhibition of the NF-ĸB pathway using si-RNA or small-molecule inhibitors can sensitize PDAC cells towards gemcitabine [14,39]. Literature reports as well as our own data showed that Panc-1 cells were rather resistant to gemcitabine treatment (Figure A3) [51,52]. The rapid and potent inhibition of NK-ĸB signaling by AS-10 (Figure 7) provided a strong rationale for combination with gemcitabine in treating PDAC, which was supported by an in vitro experiment (Figure 8) for synergistic cytotoxicity efficacy enhancement.

With respect to the unique PDAC biology of extensive stromal fibrosis, RNA-seq suggested potential inhibition of TGFβ downstream signaling in terms of the target gene expression of *TGFB1I1* and *SMAD3* without increasing ligand expression (Table S2). For further speculation of the potential side effect of AS-10 as an aspirinyl compound, RNA-seq did not reveal any suppression of the expression of multiple COX isoforms (Table S2). Therefore, AS-10 may be predicted to elicit less side effects arising from COX inhibition of common NASIDs. In vivo safety evaluation should help to address this favorably predicted benefit.

## 4. Materials and Methods

### 4.1. Synthesis and Characterization of 2-((3-(2-acetoxybenzoyl)-1,3-selenazolidin-2-ylidene)carbamoyl)phenyl Acetate (AS-10)

AS-10 was synthesized according to a newly developed methodology. Briefly, the synthesis of AS-10 was carried out by the reaction of *O*-acetylsalicyloyl chloride (1g, 5.03 mmol) with 1.12 g (7.55 mmol) of 1,3-selenazolidin-2-imine hydrobromide, obtained by the reaction in acetonitrile of 2-bromoethylamine hydrobromide (3 g, 14.64 mmol) and potassium selenocyanate (2.1 g, 14.64 mmol), in methylene chloride (50 mL) in the presence of 1.01 mL (10.06 mmol) of triethylamine. The reaction mixture was stirred at room temperature and the solid formed in the reaction (hydrohalides of triethylamine) was filtered off. Then, the solvent was removed under reduced pressure and the residue obtained was purified by chromatographic column using a gradient (from 30% to a 100% of ethyl acetate) of a mixture of hexane:ethyl acetate as a mobile phase. The purity of AS-10 (≥98%) was quantified by analytical high-performance liquid chromatography (HPLC) analysis by comparing the peak areas of the product relative to any impurities. Isolated as a white solid in a yield of 33%. m.p. = 204–207 °C. ^1^H-NMR (500 MHz, DMSO-*d_6_*) δ (ppm): 2.22 (s, 3H, OCH_3_), 2.26 (s, 3H, OCH_3_), 3.20 (t, *J* = 7 Hz, 2H, CH_2_), 4.29 (t, *J* = 7 Hz, 2H, CH_2_), 6.52 (dd, *J* = 8 Hz, *J* = 2 Hz, 1H, H_aryl_), 6.93 (td, *J* = 8 Hz, *J*=1 Hz, 1H, H_aryl_), 7.07 (dd, *J* = 8 Hz, *J* = 1 Hz, 1H, H_aryl_), 7.26 (dd, *J* = 8 Hz, *J* = 1 Hz, 1H, H_aryl_), 7.47 (td, *J* = 8 Hz, *J* = 1 Hz, 1H, H_aryl_), 7.52 (m, 1H, H_aryl_), 7.63 (m, 1H, H_aryl_), 7.67 (dd, *J* = 8 Hz, *J* = 2 Hz, 1H, H_aryl_). ^13^C-NMR (125 MHz, DMSO-*d_6_*) δ (ppm): 20.7 (1C, CH_2_), 21.0 (1C, OCH_3_), 21.4 (1C, OCH_3_), 50.3 (1C, CH_2_), 123.7 + 124.2 + 125.5 + 126.7 + 127.2 + 129.9 + 130.4 + 132.1 + 132.4 + 134.6 + 147.2 + 151.2 (12C, C_aryl_), 166.8 + 169.0 + 169.5 + 173.3 + 173.9 (5C, C=O + C=N). HRMS (ESI^+^): *m*/*z* [M+1]^+^ calcd. for C_21_H_19_O_6_N_2_Se 475.04044; found 475.04030.

Given that AS-10 is a newly synthesized compound, Lipinsky´s rule of five parameters was calculated using the free online SwissADME program (http://swissadme.ch/, accessed on 14 December 2020) as a prediction of the potential drugability of AS-10. The obtained parameters were: (i) molecular weight = 473.34; (ii) number of hydrogen bond donors = 0; (iii) number of hydrogen bond acceptors = 7; (iv) WlogP = 1.83; and (v) TPSA = 102.34 Å. Thus, no violations of any of these parameters were found, pointing towards good drugability of AS-10.

### 4.2. Reagents

A stock solution of AS-10 was prepared by dissolving in DMSO at a 10 mM concentration, storing at –20 °C. Each stock solution was used within 7 days of preparation. The final DMSO concentration for all treatments was kept at 0.1%. Antibodies were ordered from the following commercial sources: Cell signaling (Danvers, MA, USA) (IκBα (9242s), Bcl-xL (2762s), Mcl-1 (5453s) and PARP (9542s)); Abcam (Cambridge, MA, USA) (P100/P50 (ab31412), GAPDH (5174p) and P65 (ab7970)); Santa Cruz Biotechnology (Dallas, TX, USA) (P21 (sc-397); Jackson Immuno Research (West Grove, PA, USA) (Alexa fluor 680 (711-625-152) and donkey serum (017-000-002)); and Sigma-Aldrich (St. Louis, MO, USA) (β-actin (a-5441)). Rabbit IgG-chip grade protein was ordered from Abcam (ab37415), while propidium iodide (P4170-10MG), and ribonuclease A (RNAse A, R6513-10MG) and crystal violet (C6158-50G) from Sigma-Aldrich. Gemzar (gemcitabine) was obtained from Penn State Hershey Medical Center pharmacy (Hershey, PA, USA).

### 4.3. cBioPortal Database Analysis

Mutation data from the cBioPortal database (www.cbioportal.org, accessed on 14 December 2020) were downloaded [53] and the top 10 frequently mutated genes in 175 PDAC patients from The Cancer Genome Atlas (TCGA) were selected to compare common mutations with PDAC cells.

### 4.4. Cell Culture

Panc-1, BxPC3, MiaPaCa2, and mouse embryonic fibroblast (MEF) cells were maintained in DMEM medium supplemented with 10% fetal bovine serum (FBS) and 100 units/mL of penicillin and streptomycin at 37 °C and 5% CO_2_. All cell lines were obtained from American Type Culture Collection (ATCC)—MEFs—MEF (C57BL/6) [MEF-BL/6-1] (ATCC® SCRC-1008™), Panc-1—PANC-1 (ATCC® CRL-1469™), BxPC3—BxPC-3 (ATCC® CRL-1687™), and MiaPaCa2—MIA PaCa-2 (ATCC® CRL-1420™)

### 4.5. Cell Viability Assay

Briefly, 3 × 10^3^ Panc-1, MiaPaCa2, BxPC-3, or MEF cells were seeded in triplicate in a 96-well plate for 24 h and treated with DMSO as the vehicle control and different concentrations of AS-10 for the indicated time points. The MTT (3-(4,5-dimethylthiazol-2-yl)-2,5-diphenyltetrazolium) assay was performed to determine the effect of AS-10 on cancer cell viability as described in our earlier publication [22]. The IC_50_ values were determined by non-linear regression using Graphpad Prism software (La Jolla, CA, USA).

### 4.6. NAC Studies

Briefly, 1.5 × 10^4^ Panc-1 cells were seeded in a 6-well plate and allowed to adhere overnight. Cells were preincubated with NAC (5 mM, pH 7.4) for 2 h and treated with AS-10 at the indicated concentration for a period of 48 h. At the end of the 48 h, apoptosis was analyzed using the Muse Caspase 3/7 activity and Muse Annexin V assay as mentioned in the respective method sections.

### 4.7. Cell Cycle Analysis

Flow cytometry was used to determine the cell cycle phases according to our previously published method [22]. In brief, Panc-1 cells were serum starved for 72 h, followed by treatment with different concentrations of AS-10 in 10% serum containing media for different time points. The treated cells were fixed in 70% cold ethanol at the end of each time point and were stained with PI and then analyzed by a BD FACS Calibur (BD Biosciences, San Jose, CA, USA) for total DNA content as previously described [22]. Modfit software (BD Biosciences) was used to quantify the histograms.

### 4.8. Western Blot Analysis

Whole-cell lysates were made by subjecting AS-10-treated Panc-1 cells to RIPA lysis buffer (Thermo Scientific, Waltham, MA, USA) containing cocktails of protease inhibitors (Roche, Little Falls, NJ, USA) and phosphatase inhibitors (Sigma-Aldrich, USA) as described before [22]. Lysates were spun at 15,000 rpm for 10 min. The resulting supernatant was stored at −80 °C until use. NuPAGE gel 4–12% (Life Technologies, Carlsbad, CA, USA) was used to resolve the lysates (30 μg of protein per sample), followed by electro-transfer to the PVDF membrane. After the transfer, the membrane was blocked with 5% non-fat milk and probed with different antibodies. Protein of interest were detected by using an enhanced chemiluminescent reagent (Life technologies, Carlsbad, CA, USA) and X-ray films were developed using a Kodak X-OMAT 2000 processor. Blots were stripped using Restore Western blot stripping buffer (Thermo Scientific, USA) and used for loading control or other protein detection to save time and samples.

### 4.9. Caspase 3/7 Activity Assay

Panc-1 cells were treated with AS-10 at different concentrations for different time points. Caspase 3/7 activity was measured using a Muse Caspase-3/7 Assay kit with a Muse cell analyzer (EMD Millipore, Billercia, MA, USA) according to the manufacturer’s protocol and as previously described [22]. Data from the Muse cell analyzer were analyzed using Muse 1.4 software. Cells were characterized into four groups: Live (Caspase 3/7 negative, 7-AAD negative), early apoptotic (Caspase 3/7 positive, 7-AAD negative), late apoptotic (Caspase 3/7 positive, 7-AAD positive), and necrotic (Caspase 3/7 negative, 7-AAD positive).

### 4.10. Annexin V Assay

The Muse Annexin V & Dead Cell kit was used to determine the apoptotic cells in AS-10-treated Panc-1 cells as previously described [22]. In brief, Panc-1 cells were treated with a given concentration of AS-10 or DMSO for the indicated time points. Both floating cells and adherent cells were collected and subjected for Annexin V staining using a Muse Annexin V & Dead Cell kit with a Muse cell analyzer (EMD Millipore, Billerica, MA, USA). The obtained data were analyzed using Muse 1.4 software. Cells were characterized into four groups: Live (Annexin V negative, 7-AAD negative), early apoptotic (Annexin V positive, 7-AAD negative), late apoptotic (Annexin V positive, 7-AAD positive), and necrotic (Annexin V negative, 7-AAD positive).

### 4.11. Exploratory RNA Sequencing (RNA-seq), Data Processing, and Data Analysis

Total RNA from Panc-1 cells exposed to DMSO or 5 µM AS-10 for 3, 6, and 12 h was extracted using TRIzol (Thermo Fisher Scientific) and purified by column using an RNeasy Mini kit (QIAGEN). The A260/A280 ratio and RNA concentration were determined by a NanoDrop (Thermo Scientific, MA, USA). The RNA integrity number was measured by a BioAnalyzer RNA 6000 Nano Kit (Agilent, CA, USA). An A260/A280 ratio>1.9 and RIN>7 were considered acceptable. The cDNA libraries were prepared by the NEXTflex™ Illumina Rapid Directional RNA-seq Library Prep Kit (BioO Scientific) as per the manufacturer’s instructions, and unique barcode sequences were incorporated in the adaptors for multiplexed high-throughput sequencing. The final product was assessed for its size distribution and concentration by a BioAnalyzer High Sensitivity DNA Kit (Agilent, CA, USA). The libraries were pooled and diluted to 3 nM in EB buffer (Qiagen, CA, USA). The libraries were denatured, diluted to 10 pM in pre-chilled hybridization buffer, loaded onto an S1flow cell on an Illumina NovaSeq 6000, and run for 100 cycles (pair end). De-multiplexed and adapter-trimmed sequencing reads were generated using Illumina bcl2fastq (released version 2.18.0.12), allowing no mismatches in the index read. BBDuk (http://jgi.doe.gov/data-and-tools/bb-tools/, accessed on 14 December 2020) was used to trim/filter low-quality sequences using the “qtrim=lr trimq=10 maq=10” option. Next, alignment of the filtered reads to the human reference genome (GRCh38) was done using HISAT2 (version 2.1.0) [54] applying --no-mixed and --no-discordant options. Read counts were calculated using HTSeq [55] by supplementing Ensembl gene annotation (GRCh38.78).

The limma version 3.20.9 R package was used to perform quantile normalization of the log2 of the FPKM values of the reliably expressed protein coding genes, where reliably expressed protein coding genes were defined as two or more samples having FPKM >= 10, and protein coding genes were extracted by using the GENCODE Release M4 (GRCm38.p3) gene annotations. As this RNA-seq data is exploratory without replicates, the data only have exploratory value. Genes with fold change > 3.0 were considered as differentially expressed in this study. Ingenuity Pathway Analysis (IPA, QIAGEN Redwood City, www.qiagen.com/ingenuity, accessed on 14 December 2020) was used to identify canonical pathways, biological functions, upstream regulators, and top biological networks formed among the differentially expressed genes. For functional enrichment analyses of the exploratory RNA-seq data, we only selected the canonical pathways, the biological functions, and the upstream regulators meeting the significance cutoffs: Benjamini corrected *p* values < 0.05 and Z-score > 2 or < − 2 (equivalent to *p* value < 0.05). For the network analysis, IPA Molecule Activity Predictor was applied to predict the expression effects/coherence of expression effects of molecules on other network molecules.

### 4.12. Electrophoretic Mobility Shift Assay (EMSA) for NK-ĸB DNA Binding

Panc-1 cells were treated with the DMSO vehicle control or different concentrations of AS-10 for 6 h and were stimulated with or without 100 ng/mL TNF-α (3.9 nM) for an additional 30 min to determine the impact on NF-ĸB activation. At the end of 30 min, cells in the culture vessels were immediately chilled on an ice bed for the preparation of nuclear and cytosolic extracts as described previously [56]. Nuclear lysates were subjected to EMSA following the protocol [56]. The cytosolic extracts were probed by Western blot for IĸB abundance.

### 4.13. Immunocytochemistry for NF-κB P65 Localization

Panc-1 cells were seeded on top of a glass cover slip in DMEM medium and allowed to attach overnight at 37 °C in an incubator with 5% CO_2_. On the next day, cells were treated with 10 µM AS-10 for 6 h and stimulated with TNF-α (100 ng/mL, 3.9 nM) for an additional 30 min. At the end of 30 min, plates were kept on an ice bed and cells were fixed with 4% formaldehyde in PBS. Coverslips were washed using 1 × PBS, and incubated in blocking solution (0.3% Triton X-100, 5% donkey serum, made in 1 × PBS) for 1 h. Following blocking, coverslips were incubated with primary antibody solution (primary antibody diluted 1:500 in 1% BSA solution containing 0.3% Triton X-100) overnight at 4 °C in the dark. After incubation, cover slips were washed and incubated with 0.03% H_2_O_2_ for 15 min at room temperature in the dark. Secondary antibody (Alexa fluor 680) was added at a dilution of 1:500 in 1% BSA containing 0.3% triton X-100 and further incubated for 1 h. After incubation, coverslips were washed and mounted on glass slides with mounting medium containing DAPI (17985-50, Electron Microscopy Sciences, Hatfield, PA, USA). Slides were allowed to dry for 2 days and microphotographs were taken using a DELTA VISION microscope using a 60X lens. Imaris 8.2 software was used for image processing [56].

### 4.14. Live/Dead Assay with the Viability/Cytotoxicity Kit (L3224, Life Technologies, USA)

Panc-1 cells were treated with either DMSO, gemcitabine, AS-10, or in combination (AS-10 + gemcitabine) for the indicated concentrations and time points. Live and dead cells were determined by subjecting treated cells to a LIVE/DEAD Viability/Cytotoxicity Kit as previously described [22]. Stained cells were visualized using a fluorescence microscope (Zeiss—Axio Scope.A1) with a 20X objective lens. The percentage values of live and dead cells were calculated.

### 4.15. Statistical Analyses

All the values presented are expressed as mean ± SD of three independent experiments. GraphdPad Prism 8 software (La Jolla, CA, USA) was used to perform all statistical analyses. Differences among the treated groups were analyzed by ANOVA. For comparison involving only two groups, the Student t-test was used. *p* < 0.05 was considered statistically significant.

## 5. Conclusions

In summary, we identified AS-10 with selective cytotoxicity against PDAC cells while sparing the normal MEF cells. In the Panc-1 cells, AS-10 inhibited inflammatory cytokine-stimulated NF-κB signaling, arrested cell cycle progression at the G_1_ boundary, and induced caspase-mediated apoptosis with an involvement of ROS, yet without triggering necrosis or DNA damage gene signatures. The role of ROS should be further elucidated. AS-10 synergized with gemcitabine in vitro at killing PDAC cells. RNA-seq profiling informed transcriptional vs. post-transcriptional actions beside a targeting action against the eNF-κB pathway and potential inhibition of fibrosis signaling that is pervasive in PDAC. Overall, the data suggest that AS-10 may be a promising therapeutic drug lead for PDAC, both as a single agent and particularly in combination with gemcitabine. Optimization of its delivery formulations, and investigation of its pharmacokinetics and metabolism, in vivo safety, and anticancer efficacy and PD targets are warranted.

## 6. Patents

Sharma AK, Amin S, Plano D, Karelia D. Novel Selenazolidine and Thiazolidine Compounds for Treating Cancer and Other Diseases: US20170260151A1, WO/2017/160753.

## Data Availability

No new data were created or analyzed in this study. Data sharing is not applicable to this article.

## Supplementary Materials

The following are available online at https://www.mdpi.com/article/10.3390/ijms22094966/s1, Table S1: The status of the most common mutations of selected PDAC cell lines. Table S2. Select genes in G1/S cell cycle checkpoint regulation, apoptosis/survival, and NF-κB pathways in Panc-1 cells exposed to AS-10 (5 µM) vs. DMSO controls (set as 1). Table S3a: Raw RNA-seq data for Panc1 treated with AS-10 at 5 µM for 3, 6, and 12 h. Table S3b: Credible RNA-seq data for Panc1 treated with AS-10 at 5 µM for 3, 6, and 12 h (*p*-value < 0.05).

## Abbreviations

ASA	Acetyl salicylic acid, i.e., Aspirin
PDAC	Pancreatic ductal adenocarcinoma
NAC	N-acetyl cysteine
NSAID	Non-steroidal anti-inflammatory drug
PS	Phosphatidylserine
EMSA	Electro mobility shift assay
MEFs	Mouse embryonic fibroblasts
PE	Plating efficiency
GI	Gastrointestinal
PI	Propidium iodide
HDAC	Histone deacetylase
7-AAD	7-Aminoactinomycin D
HER2	Receptor tyrosine-protein kinase erbB-2
MMPs	Matrix metalloproteinases
VEGF	Vascular endothelial growth factor
EGFR	Epidermal growth factor receptor
MTT	3-(4,5-Dimethylthiazol-2-yl)-2,5-diphenyltetrazolium bromide
